# Multi-disciplinary team meetings for lung cancer in Norway and Denmark: results from national surveys and observations with MDT-MODe

**DOI:** 10.2340/1651-226X.2024.40777

**Published:** 2024-08-11

**Authors:** Anja Gouliaev, Janna Berg, Rana Bibi, Arman Arshad, Håkon Olav Leira, Kirill Neumann, Christina Aamelfot, Niels L. Christensen, Torben R. Rasmussen

**Affiliations:** a Department of Respiratory Diseases and Allergy, Aarhus University Hospital, Aarhus N, Denmark; b Department of Clinical Medicine, Aarhus University, Aarhus N, Denmark; c Department of Pulmonology, Vestfold Hospital Trust, Tønsberg, Norway; d Department of Respiratory Diseases, Aalborg University Hospital, Aalborg, Denmark; e Department of Respiratory Medicine, Odense University Hospital, Odense, Denmark; f St Olavs Hospital, Trondheim University Hospital, Trondheim, Norway; g Pulmonary Department, Akershus University Hospital, Lørenskog, Norway; h Department of Thoracic Medicine, Haukeland University Hospital, Bergen, Norway

**Keywords:** Lung cancer, Scandinavian and Nordic countries, multidisciplinary team conference, tumor board, healthcare survey, MDT-MODE, healthcare survey

## Abstract

**Background and purpose:**

Multi-disciplinary Team (MDT) meetings are widely regarded as the ‘gold standard’ of lung cancer care. MDTs improve adherence to clinical guidelines for lung cancer patients. In this study, we describe and compare lung cancer MDTs in Denmark and Norway by combining national surveys and the MDT-Metric for the Observation of Decision-making (MDT-MODe) instrument.

**Materials and method:**

Identical surveys were sent out to all lung cancer MDT centers in Denmark and Norway by the Danish Lung Cancer Group and the Norwegian Lung Cancer Group. Six MDT centers, three in Denmark and three in Norway, were observed using the MDT-MODe instrument.

**Results and interpretation:**

We found similar organization of MDT meetings in both countries, with the main difference being more local MDT meetings in Norway. All lung cancer MDTs were chaired by respiratory physicians and attended by a radiologist. Other members included oncologists, pathologists, thoracic surgeons, specialist nurses, nuclear medicine specialists and junior doctors. Overall, members reported that they had sufficient time for preparation and attending MDT meetings. With the MDT-MODe instrument it was found that the MDT chairs, surgeons, oncologists, radiologists all contributed positively to case discussion. Comorbidities were included in the discussion of most patients while the patient’s view and psychosocial issues were less often discussed. A treatment decision was reached in 79.7% of cases discussed. In conclusion, we found similar settings and overall good quality concerning lung cancer MDT meetings in Denmark and Norway.

## Introduction

Over the last two decades, multidisciplinary teams (MDTs) have become the model of care planning for patients with lung cancer worldwide. MDT meetings provide a platform for the various subspecialities to convene on a regular basis to deliberate on the diagnosis, staging and treatment of lung cancer patients. The meetings serve to ensure a timely, thorough, and focused diagnostic process and that the best available evidence-based care is offered to the patients [1–3]. MDTs improve communication, coordination and decision-making among healthcare professionals when weighing up treatment options for lung cancer patients. Cancer patients managed based on MDT decisions have been demonstrated to be more accurately staged and MDTs have been shown to improve adherence to clinical practice guidelines for lung cancer patients [4–6].

High-quality case management that supports effective MDT decision-making requires access to relevant information, structured case presentations, leadership skills and a meeting infrastructure that supports and encourages balanced contribution from team members. The MDT-Metric for the Observation of Decision-making (MDT-MODe) tool was developed as a tool for scientific assessment of MDT performance for each patient [7]. The MDT-MODe measures the quality of presented patient information, contribution to case review per specialty, and team ability to reach a decision in the MDT and has previously been used to observe MDT performances including lung cancer MDT [8–10].

Despite the emphasis on MDT meetings as a critical element in optimal decision-making in the national guidelines for diagnosing and treating lung cancer patients, the implementation of these MDT meetings has never been assessed. The Nordic countries including Denmark and Norway are similar in terms of free access to a tax-funded universal healthcare system that is provided to all citizens. However, 5-year survival for lung cancer patients is superior in Norway compared to Denmark [11]. In this study, we combine nationwide surveys for all lung cancer MDT centers in both countries by using the MDT-MODe instrument to compare MDT meetings for lung cancer in Denmark and Norway. Our primary objectives were to describe and compare the structures of MDTs in Denmark and Norway, and to assess the team decision-making during MDT meetings as differences in this important element in the decision making for the individual patient might contribute to the observed differences in survival.

## Method

### Lung cancer setting in Denmark

In Denmark, MDT meetings in lung cancer care were implemented in 2005, in order to reduce waiting times and streamline the staging process and treatment. Currently, 13 hospitals in Denmark treat lung cancer patients and, in all sites, respiratory physicians are responsible for the lung cancer MDT. Other participating specialties in the MDT meetings are radiologists, oncologists and thoracic surgeons, and at most MDT meetings also pathologists and nuclear medicine specialists. In 2022, 85.5% of lung cancer patients were discussed in MDT meetings within 90 days of referral to lung cancer work-up [12]. Waiting time for patients have indeed been reduced since 2005; to a median of 22 days between referral and start of treatment in 2022 [12].

### Lung cancer setting in Norway

In Norway, MDT meetings in lung cancer care were implemented in 2015. Currently, there are 19 lung cancer MDT sites, of which seven are ‘regional’ and 12 are ‘local’ MDTs. The MDT meetings are chaired by respiratory physicians and other participating specialties include radiologists, oncologists, pathologist and thoracic surgeons. The ‘regional’ MDT meetings cover all lung cancer patients with curative potential from the whole country (TNM stage I-III and Eastern Cooperative Oncology Group Performance Status [ECOG PS] ≤ 2) and include thoracic surgeons. In ‘local’ MDT meetings other lung cancer patients are discussed and the meetings have different and sometimes a less formal structure. There is no consensus on mandatory discussions of patients in stage IV. In 2023, 92.6% of lung cancer patients eligible for curative treatment were discussed on MDT meetings [13].

### Nationwide surveys

On behalf of the Danish Lung Cancer Group and the Norwegian Lung Cancer Group identical cross-sectional surveys were sent out by email in the fall of 2022 to all lung cancer MDT meetings in both countries. The survey aimed to evaluate the real-world implementation MDT meetings in Denmark and Norway, specifically in the context of diagnosing and treating lung cancer patients. The evaluation was based on responses from clinicians who participated in these MDT meetings. The survey, comprising 21 questions, explored various aspects of the meetings (for full survey, see supplementary material). These aspects were selected in accordance with a Danish national generic guideline for MDT meetings for cancer patients [14].

Domains covered in the survey:

Participants in the MDT meeting

Patients discussed

Educational function of the MDT meeting

Physical conditions and equipment

Time available for preparing and discussing cases

Time available for follow-up on decisions from the MDT meeting

Documentation of decisions in the patient’s medical record

Documentation of the MDT meeting in national registries

Quality assessments of decisions relative to guidelines and to other MDT meetings

If site visits to other MDT meetings for inspiration and learning had been done

Most questions in the survey were closed-ended. The survey was created using SurveyMonkey^®^ and disseminated as an online questionnaire to all MDT sites across both countries. It was sent via email to the chairperson of each MDT site, ensuring a single response per site. After a 2-week period, responses were reviewed, and reminders were sent to non-responders.

### Observations

This part of the study was designed as a prospective observational study. Six MDT teams were invited to participate in the study; three in Denmark (Odense University Hospital, Aalborg University Hospital and Aarhus University Hospital) and three ‘regional’ MDT teams in Norway (Akershus University Hospital, St. Olavs Hospital and Haukeland Hospital). All six sites responded positively at first contact and participated in this study.

Observations were conducted by one investigator (AG) in January and February 2024. We used the MDT-MODe tool to examine the MDT meetings in all six sites. MDT-MODe was developed as a tool for scientific assessment of MDT performance for each patient by Lamb et al. [7]. The MDT-MODe measures the quality of presented patient information, contribution to case review per specialty, and team ability to reach a decision in the MDT. Briefly, the MDT-MODe is a validated observational assessment instrument of quality of MDT meetings, that assesses team conduct in 13 different domains in two categories: the availability of information and the contribution of the MDT meeting participants for each patient discussed. The first part assesses presented information in six individual variables: patient case history, radiological images, histopathology, psychosocial issues, comorbidities, and patients’ views on treatment options scored on a behaviorally anchored five-point scale where five is optimal and one is insufficient. The second part assesses contributions from MDT-participants including chairpersons, surgeons, oncologists, physicians, nurse specialist, radiologists and histopathologists on a five-point scale where five is optimal and one is insufficient. Concerning comorbidity, psychosocial issues and patient view, a score of 5 is given for comprehensive first-hand knowledge, whereas comprehensive second-hand knowledge scores 3. Prior to the study, the main data collector (AG) trained in using the MDT-MODe tool at several MDT meetings at Aarhus University Hospital.

### Statistics

The responses from the survey were analyzed in Excel^®^, using fractional distribution to measure the range of answers to each question. Characteristics of MDT meetings are presented using descriptive statistics and present numbers and percentages. Results from the MDT-MODe observations are presented as means by country. Results by country were compared by Kruskall-Wallis equality-of-population rank test. Analysis of MDT-MODe was performed by using STATA^^®^^ V.18.0.

## Results

### Survey results

**Figure 1:**
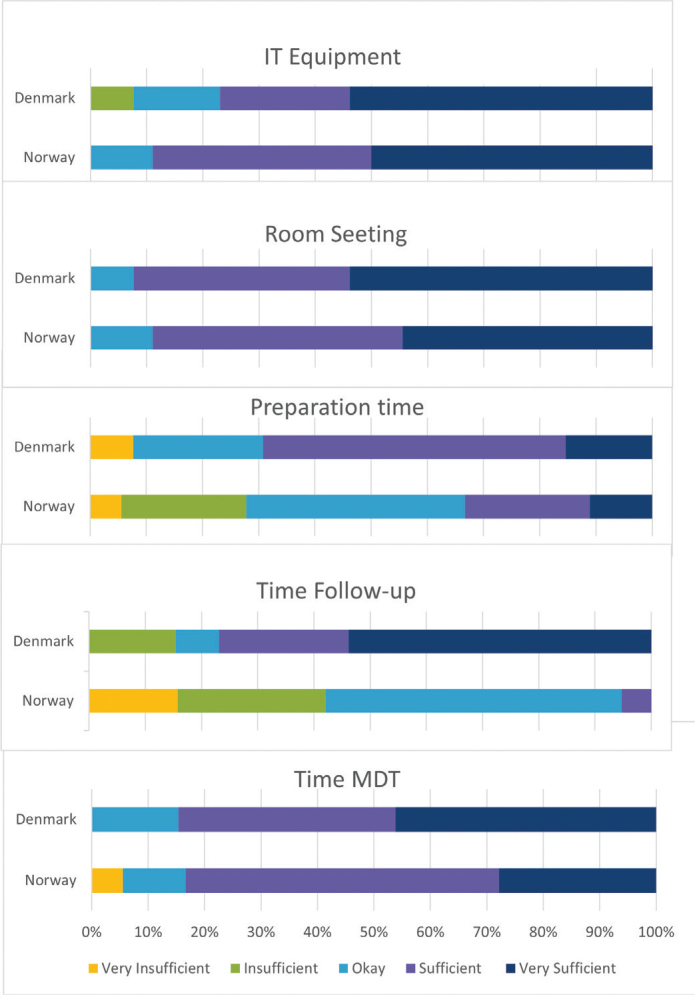
Results from national surveys on IT equipment, room/setting, preparation time, time for follow-up and allotted time for MDT meeting. Options were: very insufficient, insufficient, okay, sufficient and very sufficient.

All 13 Danish lung cancer MDT centers, all seven Norwegian ‘regional’ lung cancer MDT centers and 11 of 12 ‘local’ lung cancer MDT centers in Norway responded to the survey. Results are presented in Table 1 and Figure 1. Respiratory physicians chair all MDT meetings in both Denmark and Norway and all centers have a radiologist present. All Danish lung cancer MDTs and all seven Norwegian ‘regional’ lung cancer MDTs have a thoracic surgeon and an oncologist present. A specialist in nuclear medicine and pathology are present in 11 out of 13 (85%) lung cancer MDT meetings in Denmark, less often in Norway (44%), regardless of whether it’s a ‘local’ or ‘regional’ meeting. In the majority of MDT meetings, junior doctors are only occasionally present in Denmark, whereas they are always present in most of the ‘regional’ MDT meetings in Norway. In approximately 30% of MDT meetings, all patients are discussed. Patients not discussed are typically patients with lung cancer in non-curable stage.

**Table 1:** Results from national surveys from Danish and Norwegian centers of lung cancer MDT.

Survey Question	Denmark *N* = 13	Norway – Regional *N* = 7	Norway – Local *N* = 11
Leader of MDT	Respiratory physician	Respiratory physician	Respiratory physician
Specialties present			
Respiratory	13 (100%)	7 (100%)	11 (100%)
Radiology	13 (100%)	7 (100%)	11 (100%)
Nuclear medicine	11 (85%)	4 (57%)	4 (36%)
Pathology	11 (85%)	5 (71%)	4 (36%)
Oncology	13 (100%)	7 (100%)	8 (72%)
Thoracic surgery	13 (100%)	7 (100%)	0 (0%)
All patients discussed	4 (31%)	1 (14%)	5 (45%)
Written case presentation	13 (100%)	5 (71%)	5 (45%)
Patient preferences on MDT			
Yes	6 (46%)	3 (43%)	3 (27%)
Partially	3 (23%)	3 (43%)	6 (55%)
Junior doctors			
Yes	2 (15%)	6 (86%)	3 (27%)
Sometimes	9 (69%)	1 (14%)	6 (55%)

MDT: multi-disciplinary team.

Lung cancer MDT centers were asked about equipment and room facilities for MDT meetings (Figure 1). In both Denmark and Norway, more than half of the lung cancer MDT centers responded that their IT equipment and settings are fully sufficient to support the meetings. Five (27.8%) centers in Norway do not have sufficient time for preparation of the MDT. Only one (7.7%) of center in Denmark reports insufficient time for preparation. Actual time for conducting the MDT meetings are sufficient in both countries. The survey reveals that there is room for improvement in terms of internal and external audits, to ensure best practice according to guidelines. Similarly, very few centers have visited other lung cancer MDT centers.

### Observations

MDT-MODe observations were collected from 69 patient cases presented at the respective MDT’s, 47 from Denmark and 22 from Norwegian ‘regional’ MDT during a total of 6 MDT meetings. Table 2 shows summary of participants and number of cases at each MDT meeting. Mean time spent per case was 5 min and 5 s. Table 3 shows at which point of care the patient was discussed and Table 4 the percentage of cases, where treatment decisions were reached (76.6% in Denmark and 86.4% of cases in Norway). For all cases discussed at the meetings, a clear decision was made. But for some of the cases, the decision was to defer to the next MDT meeting, due to the need for further work-up. In the three Danish MDT meetings, both potentially curative and palliative patients were discussed. In the three ‘regional’ Norwegian MDT meetings, only potentially curative patients were discussed.

**Table 2:** Summary Data on MDT Teams Observed.

Country	MDT site	Number of participants (incl. junior doctors)	Junior doctors present	Number of case discussions observed	Duration	Mean time per case discussion
Denmark	OUH	13	Yes	11	57 m	5 m 11 s
	AAUH	11	Yes	12	56 m	4 m 40 s
	AUH	8	No	25	107 m	4 m 17 s
Norway	Akershus	14	Yes	7	25 m	3 m 34 s
	St. Olav	10	Yes	8	58 m	7 m 15 s
	Haukeland	15	Yes	7	48 m	6 m 51 s

MDT: multi-disciplinary team; OUH: Odense University Hospital; AAUH: Aalborg University Hospital; AUH: Aarhus University Hospital.

**Table 3:** A summary of point of care by country.

Country	Pre-treatment	Post-treatment	Recurrence/surveillance	Total
Denmark	29 (61.7%)	4 (8.5%)	14 (29.8%)	47
Norway	15 (68.2%)	1 (4.6%)	6 (27.3%)	22

**Table 4:** Discissions reached on MDT by country.

Country	No clear decision	Clear treatment plan	Refer to next MDT
Denmark	0	36 (76.6%)	11 (23.4%)
Norway	0	19 (86.4%)	3 (13.6%)
Total	0	55 (79.7%)	14 (20.3%)

MDT: multi-disciplinary team.

**Figure 2:**
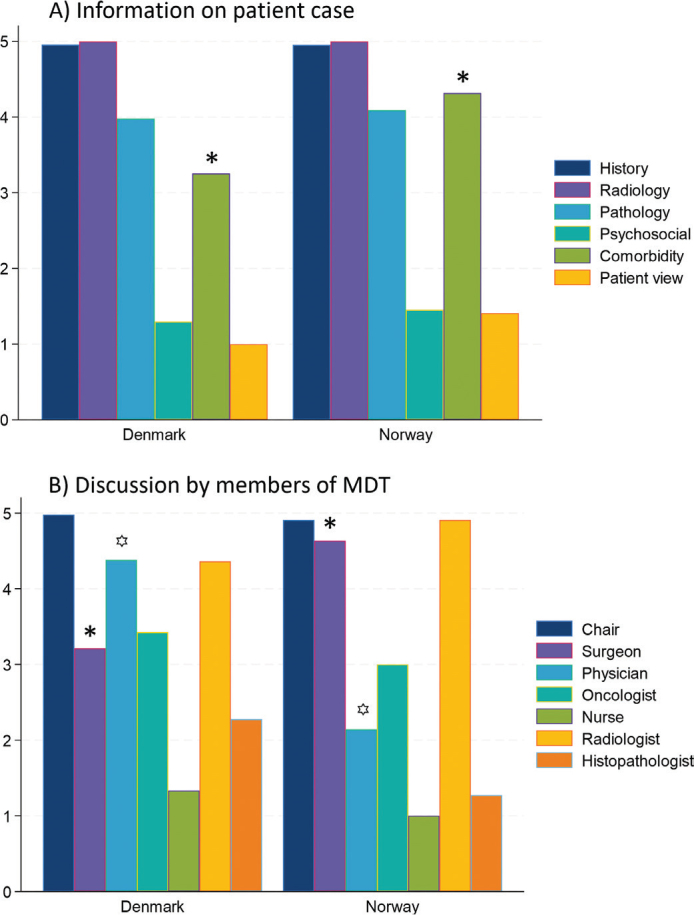
Mean score on MDT-MODe by country. Significant different values are marked with * or ꙳. *P*-value comorbidity < 0.001, surgeon 0.005 and physician < 0.001.

MDT-MODe scores on patient information are shown in Figure 2a. High scores (> 3) were seen for patient history (mean score 4.96 in Denmark, 4.95 in Norway), radiology (mean score 5 in both countries), pathology (mean score 3.98 and 4.09) and comorbidity (mean score 3.26 and 4.32). At all 6 sites, radiologists and pathologists were present and presented information on every case. For approximately 20% of cases, no biopsy/pathology had been performed (score = 1). Low scores were seen for information on psychosocial factors (mean score 1.30 and 1.45) and patient view on treatment (mean score 1 and 1.41). Scores were similar between countries, except for comorbidity and patient view. In Norway, first-hand information on comorbidity, psychosocial factors and patient view were presented more often than in Denmark (score = 5). High scores in discussion (Figure 2b) were documented for chairs (mean score 4.98 and 4.91), surgeons (mean score 3.21 and 4.64), oncologists (mean score 3.43 and 3.00) and radiologists (mean score 4.36 and 4.91). In 3 out of 6 MDT meetings, a specialist nurse was present. However, they did not participate in case discussion (mean score 1).

## Discussion

This is the first study to describe and compare lung cancer MDT meetings in Denmark and Norway. In order to compare MDT meetings for lung cancer, we applied the MDT-MODe instrument as well as national surveys to all MDT centers. We found similar settings for lung cancer MDT meetings in both countries. Furthermore, all lung cancer MDT meetings were chaired by a respiratory physician and in all meetings a radiologist presented CT scans etc. Based on survey responses, the MDT members had adequate facilities and time for MDT meetings. Measured by the MDT-MODe instrument, we found excellent case presentations regarding patient history, radiology, pathology and comorbidity. However, less frequently sufficient information on patient view and psychosocial factors. MDT members generally contributed significantly to case discussion and in most cases a treatment recommendation was reached.

While the MDT meetings in the two countries were largely similar, the national surveys also exposed some differences in lung cancer MDT structures between Denmark and Norway. In Denmark, MDT meetings are centralized, whereas in Norway there are ‘regional’ and ‘local’ MDT meetings. We found similar settings for lung cancer MDT meetings in Denmark and ‘regional’ meetings in Norway. One difference being that junior doctors are only occasionally present in Denmark, whereas they are present in most of the ‘regional’ MDT meetings in Norway, thereby utilizing the important educational potential of the MDT meeting. Even though Denmark has a larger population and more cases of lung cancer than Norway, there are more lung cancer MDT centers in Norway, thus each MDT center in Norway will discuss fewer patients. In Danish lung cancer MDTs, more specialties are represented encompassing thoracic surgery and oncology, while in Norway thoracic surgeons did not participate in all MDT meetings. Significant geographical differences also exist between the countries, with Norway often having longer distances between hospitals and in many areas a low population density compared to Denmark. In some regions of Norway, respiratory physicians administer palliative chemotherapy, while in Denmark, only oncologists are responsible for treating lung cancer patients with chemotherapy. In both countries, not all lung cancer patients are discussed on MDT meetings. However, all patients with a potential for curative treatment are discussed on MDT meetings in both countries. In the United Kingdom (UK), where MDTs have been extensively investigated, a shift toward streamlining MDT meetings, including discussing only selected patients, have been implemented [15].

In the observational part of our study, we generally found lung cancer MDT meetings to be of shorter duration and with fewer cases (7–25), compared to reports from other countries [8, 15]. Mean time spent discussing each case was around 5 min, with a trend toward shorter time spent in Danish lung cancer MDT meetings. Similar time spent per case were found in two Swedish papers with 6 min for lung cancer [16] and 5–7.9 min for rare tumors [17]. However, articles from the UK report shorter duration for each case with 3.2 min [7] and 1.5 min [18] per case discussed for cancer types other than lung. In MDT settings with +20 patients discussed, there is a risk for MDT members to experience decision making fatigue [19], however, with less than 20 cases discussed in most Danish and Norwegian lung cancer MDT meetings, this would not be an issue.

Case presentations during different MDT meetings provide an opportunity to review and re-evaluate imaging, pathology and patient-related factors based on access to relevant data and active participation from team members to achieve high-quality decision-making. From our observational study, we found high scores with the MDT-MODe instrument on case-presentation within patient history, radiology and pathology in line with results from other studies [7, 8, 17, 20]. Surgeons scored higher in the Norwegian MDT meetings compared to the Danish. But in the three MDT meetings observed in Norway, only potentially curable patients were discussed, thus, the thoracic surgeons were involved in discussions of most cases. In the Danish MDT meetings, palliative cases were also discussed, where the surgeons did not have any comment, resulting in a lower mean score.

Mean scores for discussing comorbidity were higher in this study compared to previous studies on different tumor MDT meeting [8, 9, 17, 21]. For lung cancer patients specifically, pulmonary function and cardiac comorbidities are crucial information necessary to discuss possible curative treatment (most importantly for thoracic surgery, but also for SBRT or combination chemotherapy and radiation). We report low scores on incorporating patient view and psychosocial factors in the presentation of the patient at the MDT meeting, in line with previous studies on other cancer MDTs [8, 9, 17, 21]. One reason is probably, that as fast-track lung cancer pathways in both Denmark and Norway speeds up the diagnostic work-up for lung cancer, patients often have not been informed about the lung cancer diagnosis prior to their case being reviewed in the MDT meeting. Accordingly, when patients’ cases are discussed in the MDT, data on their attitudes and preferences are frequently not known, and therefore cannot be incorporated into care management recommendations. In this study, we report low scores of input from specialist nurses, in line with other studies [8, 10, 17]. Specialist nurses are often present at the MDT meeting in order to facilitate swift follow-up or further work-up [22, 23]. We found that in 55 out of 69 cases (79.7%) a treatment recommendation was reached. In the remaining cases, more diagnostic tests were needed in order to decide on treatment. Previous studies have reported similar treatment recommendations reached; 85% in a study on urological cancers [8], 74.3% in a German study on several different cancer types [9] and 86% in British study on lung cancer patients [10].

## Strengths and limitations

This is the first study to compare lung cancer MDT meetings in Denmark and Norway by two methods; national surveys and the validated MDT-MODe instrument. We received completed survey from 96.9% of lung cancer MDT center in Denmark and Norway. However, this study also has limitations. The survey questions if the clinicians experience sufficient time for MDT presentation, MDT meeting and follow-up. However, we have not attempted to measure the extra work load that preparation of cases for an MDT meeting represent in the clinical work. Nor have we measured to what extent, a requirement for discussion on an MDT meeting before a treatment decision can be reached, may prolong the time from referral to treatment.

One researcher performed the MDT-MODe observations with no interobserver testing. As the observer is native Danish speaking, there was a potential language barrier for understanding Norwegian at the MDT meeting. However, the important observations for the MDT-MODe evaluation were not the details of the discussions at the MDT, rather who participated in the discussion and to what extent. In our study, we did not discriminate between standard or complex cases, which could have been relevant to investigate whether complex cases received increased attention with more comprehensive case presentations and case discussions.

In the observational part of the study there is a risk of the Hawthorne effect, where teams might change their usual behavior due to being observed. The Hawthorne effect is a limitation to observational studies, and in our study, MDT members were aware that they were being observed, however we believe that the effect on the presented results is limited.

## Conclusion

We report similar lung cancer MDT settings in Denmark and Norway with the main difference being that thoracic surgeons did not participate in all of the Norwegian MDT meetings due to a segregation into ‘local’ and ‘regional’ MDT meetings. For both countries recommendation for treatment was reached in 79.7% of cases. Respiratory physicians chair the meetings and radiologists present CT scans. MDT-MODe assessment revealed high score for chairs, surgeons, oncologist, radiologist and all contributed positively to case discussion. In general, junior doctors do not choose their work schedules and their supervisors should be encouraged to involve junior doctors in MDTs since it can serve as an important learning arena.

## Supplementary Material

Multi-disciplinary team meetings for lung cancer in Norway and Denmark: results from national surveys and observations with MDT-MODe

## Data Availability

The data collected for this study can be made available to others by contacting the corresponding author.
