# Supplementary material for: Multi-disciplinary team meetings for lung cancer in Norway and Denmark: results from national surveys and observations with MDT-MODe
Source: Acta Oncol. 2024 Aug 11;63:40777. doi: 10.2340/1651-226X.2024.40777 (PMC11332478; doi:10.2340/1651-226X.2024.40777)
Supplement: Multi-disciplinary team meetings for lung cancer in Norway and Denmark: results from national surveys and observations with MDT-MODe [file AO-63-40777-s1.pdf]

Supplementary material has been published as submitted. It has not been copyedited, or typeset by Acta Oncologica

Lung cancer MDT survey (translated from Danish/Norwegian)

1) Hospital where MDT is held?:____
2) Which specialty chairs (is in charge of) the lung cancer MDT?______________________
3) Mandatory participating medical specialties? – state which________________________
4) Are **all** lung cancer patients discussed? Yes/No
5) If not, which patients are **not** discussed and why?
6) Is MDT discussion systematically registered in a national coding system? Yes/No/Don't know
7) Do you have a written case presentation prior to MDT discussion? Yes/No/Partially
8) Are patient preferences systematically included in decision-making? Yes/No/Partially/Not relevant at time of MDT
9) Are junior doctors attending MDT meetings as part of their training? Yes/No/Sometimes
10) Are IT equipment sufficient for MDT? (scale 1-5, 5 = completely sufficient)
11) Are the physical room settings sufficient for MDT? (scale 1-5, 5 = completely sufficient)
12) Is it clear, who is in charge of IT equipment? Yes/No/Partially/Don't know
13) Is it clear, who is in charge of room settings? Yes/No/Partially/Don't know
14) Is there sufficient time to prepare for MDT? (scale 1-5, 5 = completely sufficient)
15) Is there sufficient time to conduct the MDT? (scale 1-5, 5 = completely sufficient)
16) How are decisions noted? Dictate during MDT, written during MDT, noted after MDT
17) Is there regular checks that the written conclusion from the MDT is correct? Yes/no
18) Is there sufficient time to follow-up on the MDT decisions? (scale 1-5, 5 = completely sufficient)
19) Are there **internal** audits that the decisions from the MDT correspond to best practise according to guidelines? Yes/No/No, but planned/Partially, but not systematically/No, but wish to establish
20) Are there **external** audits that the decisions from the MDT correspond to best practise according to guidelines, for instance by having a random sample of cases evaluated by another MDT? Yes/No/In progress
21) Are site-visits at other MDT meetings for exchange of experiences conducted? Yes/No/In progress

Supplemetary data from the MDT-MODe observations

	Scores				
**Denmark**	**1**	**2**	**3**	**4**	**5**
History	0	0	1 (2.1%)	0	46 (97.9%
Radiology	0	0	0	0	47 (100%)
Pathology	12 (25.5%)	0	0	0	35 (74.5%)
Psychosocial	40 (85.1%)	0	7 (14.9%)	0	0
Comorbidity	0	0	41 (87.2%)	0	6 (12.8%)
Patient view	47 (100%)	0	0	0	0
Chair	0	0	0	1 (2.1%)	46 (97.9%)
Surgeon	19 (40.4%)	0	4 (8.5%)	0	24 (51.1%)
Physician (n=29)	3 (10.3%)	0	3 (10.3%)	0	23 (79.3%)
Oncologist	16 (34.0%)	0	5 (10.6%)	0	26 (55.3%)
Nurse (n=24)	22 (91.7%)	0	0	0	2 (8.3%)
Radiologist	6 (12.8%)	0	2 (4.3%)	2 (4.3%)	37 (78.7%)
Pathologist	32 (68.1%)	0	0	0	15 (31.9%)

	Scores				
**Norway**	**1**	**2**	**3**	**4**	**5**
History	0	0	0	1 (4.5%)	21 (95.5%)
Radiology	0	0	0	0	22 (100%)
Pathology	4 (18.2%)	0	2 (9.1%)	9	16 (72.7%)
Psychosocial	18 (81.8%)	0	3 (13.6%)	0	1 (4.5%)
Comorbidity	0	1 (4.5%)	6 (27.3%)	0	15 (68.2%)
Patient view	19 (86.4%)	0	1 (4.5%)	1 (4.5%)	1 (4.5%)
Chair	0	0	1 (4.5%)	0	21 (95.5%)
Surgeon	2 (9.1%)	0	0	0	20 (90.9%)
Physician (n=14)	9 (64.3%)	0	2 (14.3%)	0	3 (21.4%)
Oncologist	10 (45.5%)	0	2 (9.1%)	0	10 (45.5%)
Nurse (n=14)	14 (100%)	0	0	0	0
Radiologist	0	0	1 (4.5%)	0	21 (95.5%)
Pathologist	20 (90.9%)	0	1 (4.5%)	0	1 (4.5%)

Percentages of cases scored 1-5 in each domain in Denmark and Norway. “Physician” refers to another respiratory physician besides the “Chair”. Not all MDTs had more than one participating respiratory physician.
